# Catheter Ablation Is Associated With a Decrease in Major Adverse Cardiovascular Events and All‐Cause Mortality in Patients With Atrial Fibrillation and Obstructive Sleep Apnea

**DOI:** 10.1111/jce.70152

**Published:** 2025-10-21

**Authors:** Ghassan Bidaoui, Christian Massad, Joe Abi‐Rached, Ahmed Abdelmaksoud, Eman Toraih, Michel Abou‐Khalil, Mayana Bsoul, Montaser Atasi, Amitabh Pandey, Nassir Marrouche, and other members of the TRIAD Team, Yara Menassa, Han Feng, Swati Rao, Chanho Lim, Ala' Assaf, Hadi Younes

**Affiliations:** ^1^ Tulane Research Innovation for Arrhythmia Discovery New Orleans Louisiana USA

**Keywords:** all‐cause mortality, atrial fibrillation, catheter ablation, major cardiovascular events, obstructive sleep apnea

## Abstract

**Introduction:**

Obstructive sleep apnea (OSA) is a common risk factor for the development and progression of atrial fibrillation (AF). Whether catheter ablation for AF management in patients with coexisting OSA yields survival benefit remains unclear. This study aimed to assess the association of catheter ablation with the incidence of major cardiovascular events (MACE) and all‐cause mortality in patients with AF and OSA.

**Methods:**

This was a retrospective cohort study using the TriNetX electronic health record (EHR) database, a global, federated clinical research network aggregating de‐identified patient data from multiple healthcare organizations. The study was conducted using real‐world data collected from community‐based healthcare institutions contributing to the TriNetX network. This multicenter, population‐based dataset includes both primary care and referral center data from across the United States and internationally. Adult patients diagnosed with both AF and OSA between November 2014 and November 2024 were included. Patients entered the study at the time of AF diagnosis, with follow‐up beginning 3 months later. A total of 18,324 patients were included after 1:1 propensity score matching, 9162 who underwent catheter ablation and 9162 who did not. Matching was performed based on over 30 clinical and demographic variables, including age, sex, body mass index, comorbidities, medications (e.g., anticoagulation), CPAP prescription, echocardiographic data, and laboratory values. Participants were selected using a convenience sampling method from those available in the database, with inclusion contingent on complete data and fulfillment of eligibility criteria. Data on refusals or exclusions were not available due to the nature of de‐identified secondary EHR data.

**Results:**

Over a median follow‐up of 807 days, 687 MACE events and 438 all‐cause mortality events were recorded. Catheter ablation was associated with a significantly reduced risk of MACE (HR: 0.596, 95% CI: 0.510–0.697) and all‐cause mortality (HR: 0.264, 95% CI: 0.208‐0.335). It was also associated with reduced risk of specific cardiovascular outcomes, including heart failure (HR: 0.376, 95% CI: 0.283–0.501), cerebrovascular disease (HR: 0.492, 95% CI: 0.348–0.696), and cerebral infarction (HR: 0.390, 95% CI: 0.207‐0.733).

**Conclusion:**

Catheter ablation in patients with AF and OSA is associated with a reduced risk of MACE, all‐cause mortality, heart failure, and thromboembolic events.

**Abbreviations**
AF: atrial fibrillation
CPAP: continuous positive airway pressure
HCOs: health care organizations
ICD: International Classification of Disease
MACE: major adverse cardiac event
OSA: obstructive sleep apnea

## 1 Introduction

Catheter ablation is a well‐established intervention for the restoration and maintenance of sinus rhythm in patients with atrial fibrillation (AF) [1]. However, its long‐term efficacy is influenced by multiple clinical factors, including the type and duration of AF, age, comorbidities, and atrial cardiomyopathy [1]. One such factor is obstructive sleep apnea (OSA), a highly prevalent but often underdiagnosed condition that has been implicated in AF initiation and progression [2, 3]. Prior studies have reported higher AF recurrence rates post‐ablation in OSA patients, raising concerns about the long‐term efficacy of catheter ablation in this population [2].

While the impact of OSA on rhythm control and AF recurrence has been extensively studied, the effect of catheter ablation on long‐term cardiovascular outcomes in this high‐risk population remains unclear. Given the lower rate of sinus rhythm maintenance with ablation in patients with OSA, there is uncertainty regarding the long‐term benefits of ablation on clinical hard outcomes in these patients. Beyond rhythm maintenance, the primary goal of AF management is to reduce the risk of major adverse cardiac events (MACE), heart failure, thromboembolic complications, and mortality. In this real‐world cohort study, we sought to assess the impact of catheter ablation on long‐term cardiovascular outcomes in patients with coexisting AF and OSA using data from the TriNetX research network, a multi‐institutional database aggregating real‐world electronic medical records.

## 2 Methods

### 2.1 Study Design

This study used data from TriNetX, a clinical research network that aggregates de‐identified data from electronic medical records across various health care organizations (HCOs) globally. The TriNetX database includes patient data on diagnoses, procedures, medications, and laboratory results contributed by participating HCOs. HCOs may include multiple facilities, including academic medical centers, specialty practices, and community hospitals. Data from each HCO is queried and aggregated in TriNetX.

The TriNetX network exclusively provides aggregated and de‐identified summaries from HCOs, ensuring the confidentiality of personal and health‐related data. Because of the de‐identified nature of the information, the use of TriNetX does not require ethics approval. All data in the TriNetX database complies with the Health Insurance Portability and Accountability Act (HIPAA) and US federal regulations. Additional details about the TriNetX network are available online at https://trinetx.com/data-sets-analytics/. Patient demographics and diagnoses were extracted based on the International Classification of Diseases (ICD), ninth and tenth revisions. Information on procedures, laboratory tests, and medications was also retrieved from the electronic health records.

### 2.2 Patient Population

The TriNetX database was used to identify patients with coexisting AF and OSA (ICD‐10‐CM codes I48.0 or I48.1 or I48.2 and G47.33). Patients were then stratified based on whether they underwent catheter ablation (SNOMED:233159005, CPT:93656, CPT:93657). Exclusion criteria included a prior diagnosis of cerebrovascular diseases (ICD‐10‐CM code I60‐I69), ischemic heart diseases (ICD‐10‐CM code I20‐I25), heart failure (ICD‐10‐CM code I50), cardiomyopathy (ICD‐10‐CM code I42), cardiac arrest (ICD‐10‐CM code I46), or cardiogenic shock (ICD‐10‐CM code R57.0). A comprehensive list of the ICD‐10‐CM codes used in this study is shown in Supporting Table 1. Data for the ablation group were provided by 55 participating HCOs, while data for the cohort that did not undergo ablation was provided by 76 HCOs. Patients joined the study at the time of their AF diagnosis, with follow‐up beginning 3 months later and continuing until an outcome occurred or until a maximum follow‐up of 5 years. Subgroup analysis were done based on LVEF (≥ 50% and < 50%), BMI (≥ 30 kg/m^2^ and < 30 kg/m^2^) and OSA Severity (severe defined as needing CPAP or OSA surgery, ICD10CM: Z99.89, CPT Codes: 21193, 42145,21196,21195, 21198, 21194, 21685, 21206, 42821, 42826, 94660, 42836, 21199, 42831)) looking at the effects of catheter ablation on MACE, CVA disease, TIA, ischemic heart disease, cerebral infarction, heart failure, angina pectoris, cardiomyopathy, acute MI and mortality.

### 2.3 Patient Outcomes

The primary outcome of the study was the occurrence of a major adverse cardiac event (MACE) during the 5‐year follow‐up period, which was determined using ICD‐code diagnoses. MACE included the diagnosis of transient ischemic attack and related syndromes (ICD‐10 code G45), other acute ischemic heart diseases (ICD‐10 code I24), acute myocardial infarction (ICD‐10 code I21), subsequent ST‐elevation or non‐ST elevation myocardial infarction (ICD‐10 code I22), ischemic cardiomyopathy (ICD‐10 code I25.5), angina pectoris (ICD‐10 code I20), heart failure (ICD‐10 code I50), cardiomyopathy (ICD‐10 code I42), cardiac arrest (ICD‐10 code I46), cerebral infarction (ICD‐10 code I63), occlusion and stenosis of precerebral arteries not resulting in cerebral infarction (ICD‐10 code I65), occlusion and stenosis of cerebral arteries not resulting in cerebral infarction (ICD‐10 code I66), and shock (ICD‐10 code R57). Further analysis was also conducted to analyze specific secondary outcomes including cerebrovascular disease (ICD‐10 code I60‐I69), transient ischemic attack (ICD‐10 code G45), ischemic heart disease (ICD‐10 code I20‐I25), cerebral infarction (ICD‐10 code I63), angina pectoris (ICD‐10 code I20), myocardial infarction (ICD‐10 code I21 and I22), cardiomyopathy (ICD‐10 code I42), heart failure (ICD‐10 code I50), and all‐cause mortality.

### 2.4 Statistical Analysis

All statistical analyses were performed using the TriNetX online research platform. Chi‐square analysis and independent sample t‐tests were used to compare categorical variables and continuous variables, respectively. Patients were propensity‐score matched in a 1:1 ratio based on the following variables: age at index, sex, race, hypertension, other cardiac arrhythmias, problems related to housing and economic circumstances, problems related to education and literacy, problems related to employment and unemployment, occupational exposure to risk factors, diabetes mellitus, metabolic disorders, obesity, hyperlipidemia, nicotine dependence, alcoholic related disorders, acute kidney failure, chronic kidney disease, chronic obstructive pulmonary disease, continuous positive airway pressure (CPAP) prescription, cardiac troponin I, c‐reactive protein, body mass index(BMI), systolic Blood Pressure, diastolic Blood Pressure, creatinine, hemoglobin, triglyceride, high‐density lipoproteins (HDL), low‐density lipoproteins (LDL), and left ventricular ejection fraction. A comprehensive list of the variables used for propensity‐score matching is provided in Supporting Table 2. Cox regression analysis was used to calculate the hazard ratios and confidence intervals for the primary and secondary outcomes of interest.

## 3 Results

### 3.1 Baseline Patient Characteristics Before and After Propensity‐Score Matching

Before propensity‐score matching, a total of 100,307 patients with coexisting AF and OSA were included in the analysis. Of these, 9412 underwent catheter ablation. Patients in the ablation cohort were younger on average (61.9 vs 64.7, *p* < 0.001) and more likely to be male (66% vs 59.8%, *p* < 0.001). Additional baseline characteristics of the cohorts before propensity score matching are included in Table 1. After propensity score matching with a 1:1 ratio based on age, sex, comorbidities, laboratory tests, and medications, the cohort included a total of 18,324 patients that were evenly divided into two groups of 9162 patients. The baseline characteristics of the propensity score matched cohorts are presented in Table 2.

**Table 1:** Baseline characteristics before propensity score matching.

Variable	AF + OSA+ ablation (*n* = 9412)	AF + OSA+ no ablation (*n* = 90,895)	*P*
**Demographics**			
Age at Index	61.9 (10.2)	64.7 (12.1)	<0.001
White	7856 (83.5%)	72,428 (79.7%)	<0.001
Male	6212 (66.0%)	54,371 (59.8%)	<0.001
Female	2761 (29.3%)	32,487 (35.7%)	<0.001
Black or African American	317 (3.4%)	6273 (6.9%)	<0.001
Hispanic or Latino	207 (2.2%)	2617 (2.9%)	<0.001
Asian	199 (2.1%)	1565 (1.7%)	0.006
Other Race	131 (1.4%)	2203 (2.4%)	<0.001
**Comorbidities**			
Hypertensive diseases	5862 (62.3%)	54,042 (59.5%)	<0.001
Metabolic disorders	5523 (58.7%)	44,414 (48.9%)	<0.001
Lipoprotein disorders/lipidemias	5083 (54.0%)	44,414 (48.9%)	<0.001
Obesity/overnutrition	4013 (42.6%)	32,625 (35.9%)	<0.001
Diabetes mellitus	1590 (16.9%)	19,723 (21.7%)	<0.001
Other cardiac arrhythmias	3257 (34.6%)	18,301 (20.1%)	<0.001
CKD/acute kidney failure	731 (7.8%)	10,714 (11.8%)	<0.001
COPD	366 (3.9%)	6894 (7.6%)	<0.001
**Social factors**			
Nicotine dependence	704 (7.5%)	6830 (7.5%)	0.904
Alcohol‐related disorders	375 (4.0%)	3018 (3.3%)	0.001
Housing/economic problems	41 (0.4%)	401 (0.4%)	0.938
Employment/unemployment	27 (0.3%)	201 (0.2%)	0.202
Occupational exposure	12 (0.1%)	94 (0.1%)	0.494
Education/literacy problems	10 (0.1%)	56 (0.1%)	0.108
**Laboratory parameters**			
Systolic BP	126.7 (17.7)	129.2 (18.3)	<0.001
Diastolic BP	76.8 (11.4)	75.4 (11.6)	<0.001
BMI	33.3 (6.6)	34.4 (8.2)	<0.001
Creatinine	1.0 (0.4)	1.0 (1.3)	<0.001
BUN	17.1 (5.3)	17.8 (8.4)	<0.001
GFR (MDRD)	76.1 (18.4)	76.4 (23.8)	0.29
Hemoglobin	14.4 (1.6)	13.7 (2.0)	<0.001
Triglycerides	132.0 (81.8)	137.7 (119.1)	0.002
HDL cholesterol	46.2 (19.3)	46.7 (18.1)	0.077
LDL cholesterol	100.6 (33.7)	98.2 (34.0)	<0.001
CRP	15.5 (37.2)	25.2 (50.6)	<0.001
Troponin I	0.1 (0.9)	0.1 (0.9)	0.614
BNP	172.2 (260.6)	204.8 (804.6)	0.209
**Echocardiography**			
LV ejection fraction (%)	59.7 (8.1)	60.4 (9.2)	0.006
LA diameter (mm)	34.0 (15.0)	31.7 (15.3)	0.007
**Procedures**			
CPAP	342 (3.6%)	2514 (2.8%)	<0.001
**Medications**			
Anticoagulants	8007 (85.1%)	45,563 (50.1%)	<0.001
Beta blockers	6789 (72.1%)	45,679 (50.3%)	<0.001
Antiarrhythmics	6789 (72.1%)	45,679 (50.3%)	<0.001
Apixaban	5677 (60.3%)	21,704 (23.9%)	<0.001
Antilipemic agents	4105 (43.6%)	34,040 (37.4%)	<0.001
Calcium channel blockers	4250 (45.2%)	30,021 (33.0%)	<0.001
Lidocaine	4451 (47.3%)	34,081 (37.5%)	<0.001
Diuretics	2928 (31.1%)	28,486 (31.3%)	0.647
Rivaroxaban	2390 (25.4%)	10,467 (11.5%)	<0.001
Flecainide	2591 (27.5%)	6264 (6.9%)	<0.001
ACE inhibitors	2022 (21.5%)	18,549 (20.4%)	0.014
ARBs	2030 (21.6%)	16,308 (17.9%)	<0.001
Heparin	1830 (19.4%)	14,442 (15.9%)	<0.001
Amiodarone	1360 (14.4%)	4763 (5.2%)	<0.001
Enoxaparin	1396 (14.8%)	12,653 (13.9%)	0.015
Warfarin	745 (7.9%)	7996 (8.8%)	0.004
Sotalol	647 (6.9%)	2266 (2.5%)	<0.001
Dronedarone	659 (7.0%)	1483 (1.6%)	<0.001
Digitalis glycosides	379 (4.0%)	2803 (3.1%)	<0.001
Dabigatran	382 (4.1%)	1913 (2.1%)	<0.001
Quinidine	10 (0.1%)	24 (0.0%)	<0.001

**Table 2:** Baseline characteristics after propensity score matching.

Variable	AF + OSA+ ablation (*n* = 9162)	AF + OSA+ no ablation (*n* = 9162)	*P*
**Demographics**			
Age at Index	62.0 (10.1)	62.0 (12.3)	0.98
White	7642 (83.4%)	7700 (84.0%)	0.246
Male	6014 (65.6%)	6058 (66.1%)	0.493
Female	2718 (29.7%)	2691 (29.4%)	0.662
Black or African American	313 (3.4%)	304 (3.3%)	0.712
Hispanic or Latino	203 (2.2%)	197 (2.2%)	0.762
Asian	187 (2.0%)	179 (2.0%)	0.673
Other Race	130 (1.4%)	118 (1.3%)	0.443
**Comorbidities**			
Hypertensive diseases	5712 (62.3%)	5647 (61.6%)	0.323
Metabolic disorders	5360 (58.5%)	5207 (56.8%)	0.022
Lipoprotein disorders/lipidemias	4930 (53.8%)	4780 (52.2%)	0.026
Obesity/overnutrition	3861 (42.1%)	3744 (40.9%)	0.079
Diabetes mellitus	1588 (17.0%)	1482 (16.2%)	0.131
Other cardiac arrhythmias	3103 (33.9%)	3061 (33.4%)	0.511
CKD/acute kidney failure	721 (7.9%)	696 (7.6%)	0.489
COPD	361 (3.9%)	338 (3.7%)	0.375
**Social factors**			
Nicotine dependence	682 (7.4%)	682 (7.4%)	<0.001
Alcohol‐related disorders	358 (3.9%)	351 (3.8%)	0.789
Housing/economic problems	40 (0.4%)	37 (0.4%)	0.723
Employment/unemployment	26 (0.3%)	24 (0.3%)	0.777
Occupational exposure	11 (0.1%)	10 (0.1%)	0.827
Education/literacy problems	10 (0.1%)	10 (0.1%)	1
**Laboratory parameters**			
Systolic BP	126.8 (17.7)	128.3 (18.1)	<0.001
Diastolic BP	76.7 (11.4)	75.9 (11.1)	<0.001
BMI	33.3 (6.6)	34.3 (7.9)	<0.001
Creatinine	1.0 (0.4)	1.0 (0.7)	0.304
BUN	17.1 (5.3)	17.0 (6.5)	0.156
GFR (MDRD)	76.0 (18.4)	78.6 (21.3)	<0.001
Hemoglobin	14.4 (1.6)	14.1 (1.9)	<0.001
Triglycerides	131.8 (81.0)	136.4 (106.8)	0.025
HDL cholesterol	46.4 (19.3)	46.1 (17.9)	0.486
LDL cholesterol	100.5 (33.8)	99.6 (33.3)	0.222
CRP	15.2 (36.7)	21.3 (45.0)	0.001
Troponin I	0.1 (0.9)	0.0 (0.3)	0.123
BNP	171.3 (253.1)	203.9 (516.6)	0.081
**Echocardiography**			
LV ejection fraction (%)	59.8 (8.2)	60.1 (8.5)	0.278
LA diameter (mm)	34.2 (15)	33.5 (14.2)	0.557
**Procedures**			
CPAP	323 (3.5)	294 (3.2)	0.235
**Medications**			
Anticoagulants	7757 (84.7%)	7754 (84.6%)	0.951
Beta blockers	6572 (71.7%)	6445 (70.3%)	0.039
Antiarrhythmics	6443 (70.3%)	6372 (69.5%)	0.253
Apixaban	5450 (59.6%)	5553 (60.6%)	0.12
Antilipemic agents	4012 (43.8%)	3921 (42.8%)	0.175
Calcium channel blockers	4106 (44.8%)	4050 (44.2%)	0.405
Lidocaine	4308 (47.0%)	4262 (46.5%)	0.496
Diuretics	2876 (31.4%)	2851 (31.1%)	0.69
Rivaroxaban	2281 (24.9%)	2322 (25.3%)	0.485
Flecainide	2392 (26.1%)	2314 (25.3%)	0.187
ACE inhibitors	1983 (21.6%)	1997 (21.8%)	0.802
ARBs	1986 (21.7%)	1996 (21.8%)	0.858
Heparin	1779 (19.4%)	1813 (19.8%)	0.527
Amiodarone	1264 (13.8%)	1274 (13.9%)	0.831
Enoxaparin	1361 (14.9%)	1342 (14.6%)	0.692
Warfarin	735 (8.0%)	716 (7.8%)	0.603
Sotalol	609 (6.6%)	633 (6.9%)	0.481
Dronedarone	605 (6.6%)	608 (6.6%)	0.929
Digitalis glycosides	369 (4.0%)	359 (3.9%)	0.705
Dabigatran	364 (4.0%)	379 (4.1%)	0.574
Quinidine	10 (0.1%)	10 (0.1%)	1

### 3.2 Ablation Effect on MACE, All‐Cause Mortality, Heart Failure

**Figure 1:**
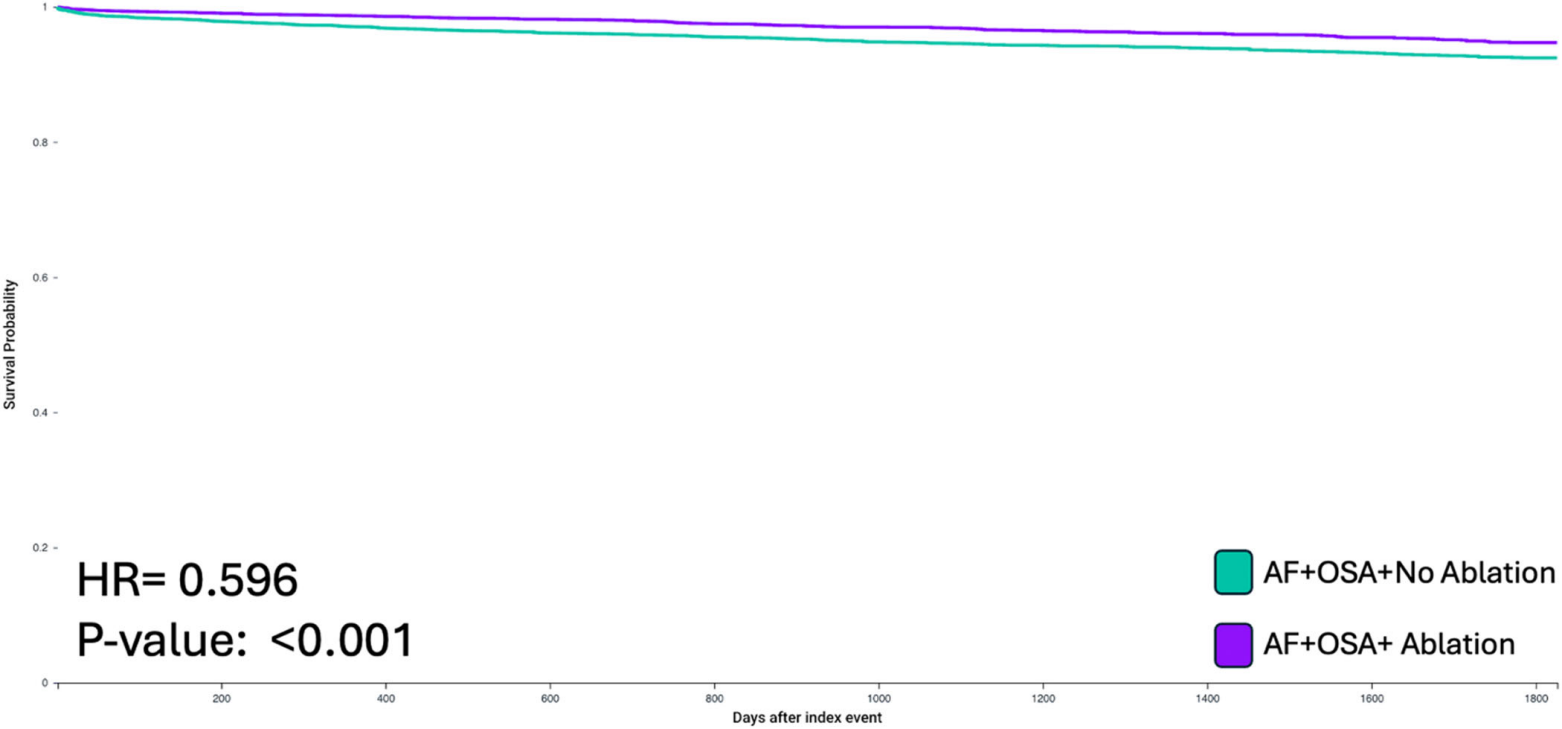
Kaplan‐Meier survival curve demonstrating decreased risk of MACE in patients with co‐existing AF and OSA who underwent Catheter Ablation. (HR, Hazard Ratio, AF, Atrial Fibrillation; OSA, Obstructive Sleep Apnea).

**Figure 2:**
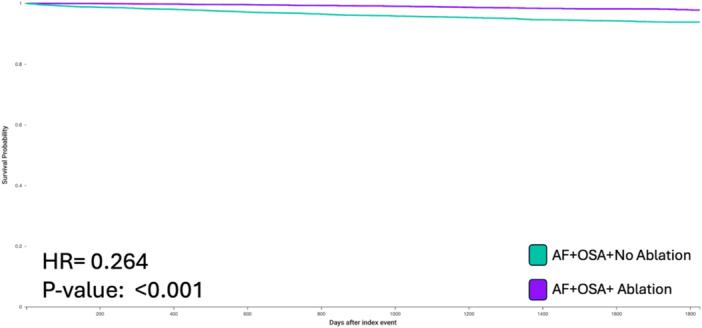
Kaplan‐Meier survival curve demonstrating decreased risk of all‐cause mortality in patients with co‐existing AF and OSA who underwent catheter ablation. (HR, Hazard Ratio; AF, Atrial Fibrillation; OSA, Obstructive Sleep Apnea).

Over a median follow‐up of 807 days, catheter ablation in patients with OSA and AF was associated with a significantly reduced risk of MACE (HR: 0.596, CI: 0.590–0.697, *p* < 0.001) (Figure 1), all‐cause mortality (HR: 0.264, CI: 0.208–0.337, *p* < 0.001) (Figure 2), and heart failure (HR: 0.376, 95% CI: 0.283–0.501, *p* < 0.001) compared to patients who did not undergo ablation.

### 3.3 Ablation Effect on Thromboembolic and Ischemic Events

Patients who underwent ablation exhibited a lower incidence of thromboembolic outcomes including cerebral infarction (HR:0.390, 95% CI: 0.207–0.733, *p* = 0.002), and cerebrovascular disease (HR: 0.492, 95% CI: 0.348–0.696, *p* < 0.001). However, catheter ablation was not associated with TIA incidence (*p* = HR 0.822, CI: 0.619–1.019, *p* = 0.174)

Catheter ablation was also associated with reduced risk of ischemic heart disease (HR: 0.581, CI: 0.469‐.719, *p* < 0.001) and myocardial infarction (HR: 0.454, CI: 0.238–0.864, *p* = 0.014). However, ablation was not associated with angina (HR: 0.882, CI: 0.448–1.737, *p* = 0.717) and cardiomyopathy (HR: 0.649, CI: 0.399–1.053, *p* = 0.078).

### 3.4 Subgroup Analysis Results

#### 3.4.1 LVEF Subgroup

The results showed that in patients with preserved EF ( ≥ 50%) before ablation, catheter ablation is associated with a decreased risk of MACE (HR 0.826, CI: 0.0.727, 0.939), morality (HR 0.219, CI:0.146–0.328), CVA disease (HR 0.75, CI: 0.589, 0.956), cerebral infarction (HR 0.585, CI: 0.367, 0.932), heart failure (HR 0.782, CI: 0.662–0.923). As for the group with a reduced LVEF at baseline (<50%), catheter ablation was associated with a decreased risk of mortality (HR 0.465, CI: 0.221–0.977), and cerebrovascular disease (HR 0.703, CI: 0.453–1.092). All other outcomes were not significantly different between the two groups.

#### 3.4.2 BMI Subgroup

In patients with elevated BMI (≥30 kg/m^2^), catheter ablation showed significant benefit for MACE (HR 0.749, CI: 0.665–0.844), CVA disease (HR 0.633, CI: 0.499–0.802), TIA (HR 0.631, CI: 0.464–0.858), ischemic heart disease (HR 0.741, CI: 0.645–0.852), cerebral infarction (HR 0.457, CI: 0.313–0.668), heart failure (HR 0.815, CI: 0.695–0.955), acute MI (HR 0.612, CI: 0.427–0.877), ischemic cardiomyopathy (HR 0.268, CI: 0.206–0.348), and mortality (HR 0.268, CI: 0.206–0.348). For those with a low BMI (< 30 kg/m^2^), catheter ablation was associated with a decreased risk of CVA disease (HR 0.775, CI: 0.628–0.956), mortality (HR 0.306, CI: 0.231–0.407), and cerebral infarction (HR 0.634, CI: 0.443–0.905). There was an association with an increased risk of cardiomyopathy in these patients (HR 1.260, CI: 1.038, 1.529). Remaining outcomes were non‐significant between both groups.

#### 3.4.3 OSA Severity Subgroup

For those with severe OSA (On CPAP or underwent OSA Surgery), catheter ablation was associated with a decreased risk of MACE (HR 0.382, CI: 0.258–0.566), mortality (HR 0.328, CI: 0.194–0.555), ischemic heart disease (HR 0.423, CI: 0.237–0.754). For patients with non‐severe disease, catheter ablation showed an association with decreased risk of MACE (HR 0.621, CI: 0.509–0.758), mortality (HR 0.271, CI: 0.197–0.372), CVA disease (HR 0.414, CI: 0.271–0.635), ischemic heart disease (HR 0.630, CI: 0.482–0.824), and heart failure (HR 0.416, CI: 0.286–0.605). There were no significant differences in the remaining outcomes.

## 4 Discussion

In this real‐world cohort study of 18,324 propensity‐matched patients with coexisting AF and OSA, we found that catheter ablation was associated with a significant reduction in MACE, all‐cause mortality, and heart failure compared to medical therapy. Additionally, ablation was linked to a lower incidence of thromboembolic events, including cerebral infarction and cerebrovascular disease, but showed no significant effect on transient ischemic attack, angina, or cardiomyopathy. These findings suggest that catheter ablation may confer long‐term cardiovascular benefits in patients with OSA and AF, despite the known challenges in maintaining sinus rhythm in this population.

OSA is a well‐established yet often underrecognized driver of AF development and progression. It is characterized by recurrent obstructive episodes, leading to intermittent hypoxia, hypercapnia, sympathetic activation, and autonomic instability [4]. These physiological disturbances contribute to cardiovascular remodeling, particularly affecting the left atrium, a critical substrate for AF [5, 6, 7]. Left atrial myopathy, defined as structural, functional, or electrical abnormalities of the left atrium, is a key driver of AF development and persistence. Given that OSA promotes atrial myopathy on a chronic, night‐to‐night basis, it has been hypothesized and proven that OSA could reduce the efficacy of catheter ablation for maintaining sinus rhythm [8]. However, our study demonstrates that despite these concerns, ablation in patients with OSA is associated with significant improvements in clinical outcomes, suggesting that its benefits extend beyond rhythm control alone.

The mechanisms underlying these favorable outcomes are likely multifactorial. Prior studies have shown that catheter ablation prolongs sinus rhythm maintenance compared to medical therapy, and maintaining sinus rhythm is associated with a lower risk of stroke and mortality in AF patients [9]. AF burden, a measure of the cumulative time a patient spends in AF, has been strongly linked to increased risk of heart failure development, a phenomenon known as arrhythmia‐induced cardiomyopathy [10]. Although OSA has been associated with lower ablation success rates in some studies, ablation still appears to confer a relative advantage in reducing AF burden compared to medical therapy alone [11]. This reduction in AF burden may partly explain the observed improvements in MACE, heart failure, and thromboembolic events in our study.

Another potential explanation is that ablation leads to favorable atrial remodeling, independent of whether sinus rhythm is fully maintained. Pulmonary vein isolation (PVI), the cornerstone of AF ablation, not only targets the primary AF triggers but also influences left atrial structure and function [12]. Studies have shown that ablation can reduce left atrial dilation, a key marker of atrial myopathy, and improve left atrial hemodynamics, including left atrial emptying fraction and atrial strain across all phases of function (reservoir, conduit, and booster phases) [13, 14]. These improvements may reduce pulmonary hypertension, a frequent consequence of OSA, thereby mitigating right ventricular and atrial remodeling [15]. Additionally, enhanced left atrial function could optimize left ventricular filling, reducing ventricular workload and hypertrophy, ultimately lowering the incidence of heart failure [15].

A particularly important finding in our study is the reduction in thromboembolic risk in patients with OSA and AF who underwent ablation. Both OSA and AF are independent risk factors for stroke, and their coexistence has been shown to have a synergistic effect on thromboembolism risk [16, 17, 18]. OSA is associated with a high prevalence of hypertension, the leading risk factor for ischemic and hemorrhagic strokes, while AF is responsible for approximately 30% of embolic strokes [19, 20]. In our propensity‐matched cohort, approximately 85% of patients were on anticoagulation in both arms, suggesting that the observed reduction in thromboembolic events is likely attributable to the ablation intervention itself. Ablation improves left atrial function, thereby reducing blood stasis and the risk of thrombus formation in the left atrial appendage [21]. This is supported by the results of OPTION trial, which demonstrated a lower incidence of stroke in patients undergoing ablation compared to historical AF cohorts [22]. Furthermore, ablation may attenuate the synergistic effect of OSA and AF on stroke risk by modulating autonomic tone and, hence decreasing autonomic instability [23].

In addition to being linked to lower thromboembolic risk, catheter ablation was also associated with a reduction in all‐cause mortality. Previous clinical trials in AF cohorts have similarly found that catheter ablation reduces all‐cause mortality. For example, the CASTLE‐AF trial found that catheter ablation reduced the risk of all‐cause mortality by 47% in patients with AF and heart failure with reduced ejection fraction [24] and the CABANA treatment‐received analysis found a 40% reduction in the ablation group in AF patients [25]. One meta‐analysis of observational studies comparing ablation and medical therapy found a 61% mortality risk reduction in the ablation group [26]. In this present study, the reduction in all‐cause mortality risk in the ablation group was 74%, which is notably larger than the effect size of previous studies. There are several explanations for this disparity. First, this study was done with a real‐world population, which differs from clinical trial populations such as those in CASTLE‐AF or CABANA. Second, our cohort is composed of patients with both AF and OSA, while the CASTLE‐AF cohort was composed of AF and heart failure patients, and CABANA was done in AF only patients. Lastly, although we attempted to control for many confounding variables, there may be some residual confounding given the retrospective nature of this study. For example, healthier, better‐supported patients tend to be referred more often for procedural interventions, while frailer patients are more likely to remain on medical therapy. Therefore, it is possible that the reduction in mortality risk in this study is exaggerated by certain residual confounding variables such as patient selection. Regardless, future prospective trials are needed to confirm these findings.

Subgroup analyses were conducted based on LVEF, BMI and severity of OSA. Catheter ablation was association with a reduction in the mortality risk regardless of baseline LVEF and lowered in MACE events in those with a normal LVEF. Both high‐ and low‐BMI patients showed an association with decreased mortality risk with ablation; however, a decrease in MACE risk was observed only in those with high BMI. In contrast, patients with low BMI showed an association with increased risk of ischemic cardiomyopathy following ablation. Ablation was also associated with a reduced risk of both mortality and MACE regardless of baseline OSA severity. Taken together, these findings support catheter ablation as a reasonable treatment strategy for patients with AF and OSA, irrespective of LVEF, BMI, or OSA severity.

## 5 Limitations

While our study provides valuable insights into the impact of catheter ablation in patients with coexisting AF and OSA, several limitations should be acknowledged. First, as a retrospective observational study, our findings are subject to selection bias and unmeasured confounders, limiting the ability to establish direct causality. For example, healthier and better‐supported patients tend to be referred more often for procedural interventions such as catheter ablation, while frailer patients are more likely to remain on medical therapy. It is possible that these unmeasured factors may have confounded the results of this retrospective study. However, the use of propensity‐score matching helped minimize baseline differences between groups, strengthening the reliability of our conclusions and warranting future study. Second, our dataset lacked information on OSA severity and continuous positive airway pressure (CPAP) adherence, both of which are known to influence AF progression and ablation outcomes. The absence of these variables limits our ability to assess whether CPAP therapy modified the observed benefits of ablation. Third, we could not account for variability in ablation techniques or operator expertise, which may impact long‐term outcomes. Nonetheless, the real‐world nature of our data reflects heterogeneous clinical practices, increasing the generalizability of our findings. Additionally, our study did not include post‐ablation AF burden assessment or detailed echocardiographic parameters, such as left atrial strain or fibrosis quantification, which could provide mechanistic insights into the observed benefits of ablation. The increased risk of nonischemic cardiomyopathy seen in the ablation group remains an area of interest, but whether this represents a true adverse effect of ablation, or an artifact of longer survival and extended follow‐up is unclear. Despite these limitations, our study leverages a large, multicenter dataset with real‐world patient data, providing strong external validity and a broad representation of patients with OSA and AF across different healthcare settings.

## 6 Conclusion

Catheter ablation is associated with a reduction in the risk of MACE and all‐cause mortality in patients with coexisting AF and OSA. Over a median follow‐up of 807 days, catheter ablation was associated with improved cardiovascular outcomes, including reducing the risk of heart failure, cerebrovascular disease, and cerebral infarction. This study supports the integration of catheter ablation into treatment protocols for patients with coexisting AF and OSA to reduce cardiovascular risk and mortality.

## Members of the TRIAD Team

Yara Menassa MD^a^, Han Feng PhD^a^, Swati Rao MD^a^, Chanho Lim BSC^a^, Ala′ Assaf MD^a^, Hadi Younes MD^a^

^a^Tulane Research Innovation for Arrhythmia Discovery, New Orleans, Louisiana, USA.

## Conflicts of Interest

N.M. reports having received consulting fees from Biosense Webster, Boston Scientific and AtriCure. N.M. also reports being a speaker for Abbott, Biosense Webster, AtriCure and Sanofi. N.M. also reports receiving research support Abbott, Medtronic, Biosense Webster, Siemens, GE, Boston Scientific, Sanofi, and Samsung. N.M. also reports having a family member as the CEO of Cardiac Designs. N.M. also reports being the founder of Marrek, being named in a patent issued for MRI fibrosis imaging and being a previous shareholder of Cardiac Designs. The other authors declare no conflicts of interest.

## Supporting information


**Supplementary Table 1:** ICD‐10‐CM codes used for diagnosis of comorbidities and outcomes. S**upplementary Table 2:** Variables included in propensity score. S**upplementary Table 3:** Incidence, hazard ratios and p‐values for outcomes of interest. MACE, major adverse cardiovascular events; TIA, transient ischemic attack; MI, myocardial infarction. **Supplementary Figure 1:** Propensity score density function ‐ Before and after matching (cohort 1 ‐ purple, cohort 2 ‐ green).

## Data Availability

The data that support the findings of this study are available from TriNetX. Restrictions apply to the availability of these data, which were used under license for this study. Data are available from https://trinetx.com with the permission of TriNetX.
