# Supplementary material for: Catheter Ablation Is Associated With a Decrease in Major Adverse Cardiovascular Events and All‐Cause Mortality in Patients With Atrial Fibrillation and Obstructive Sleep Apnea
Source: J Cardiovasc Electrophysiol. 2025 Oct 21;37(1):36–45. doi: 10.1111/jce.70152 (PMC12794737; doi:10.1111/jce.70152)
Supplement: Supplementary file 1 — Supplementary Table 1: ICD‐10‐CM codes used for diagnosis of comorbidities and outcomes. Supplementary Table 2: Variables included in propensity score. Supplementary Table 3: Incidence, hazard ratios and p‐values for outcomes of interest. MACE, major adverse cardiovascular events; TIA, transient ischemic attack; MI, myocardial infarction. Supplementary Figure 1: Propensity score density function ‐ Before and after matching (cohort 1 ‐ purple, cohort 2 ‐ green). [file JCE-37-36-s001.docx]

**Supplementary Table 1.** ICD-10-CM codes used for diagnosis of comorbidities and outcomes

| Diagnosis | ICD-10-CM Code |
| --- | --- |
| Acute coronary disease | I24 |
| Atrial Fibrillation | I48.0  I48.1  I48.2 |
| Angina pectoris | I20 |
| Cardiac arrest | I46 |
| Cardiogenic shock | R57.0 |
| Cardiomyopathy | I42 |
| Cerebral infarction | I63 |
| Cerebrovascular Disease | I60-I69 |
| Heart Failure | I50 |
| Ischemic heart disease | I20-I25 |
| Major Adverse Cardiovascular Events | G45  I24  I21  I22  I25.5  I20  I63  I65  I66  R57 |
| Myocardial infarction | I21  I22 |
| Obstructive Sleep Apnea | G47.33 |
| Transient Ischemic Attack | G45 |

S**upplementary Table 2.** Variables included in propensity score

| Demographics | Age at Index  Sex  Race |
| --- | --- |
| Diagnoses | Hypertension  Other cardiac arrhythmias  Problems related to housing and economic circumstances  Problems related to education and literacy  Problems related to employment and unemployment  Occupational exposure to risk factors  Diabetes Mellitus  Metabolic Disorders  Obesity  Hyperlipidemia  Nicotine dependence  Alcoholic related disorders  Acute kidney failure  Chronic kidney disease  Chronic obstructive pulmonary disease |
| Echocardiographic data | Left ventricular ejection fraction (%) |
| Laboratory tests[mass/volume] in serum, plasma, or blood | Troponin I. Cardiac  C reactive protein  BMI  Systolic Blood Pressure  Diastolic Blood Pressure  Creatinine  Hemoglobin  Triglyceride  HDL  LDL |
| Medications | Amiodarone  ACE inhibitors  Beta blockers  Diuretics  Calcium channel blockes  Antilipemic agents  Angiotensin II inhibitors  Digitalis glycosides  Quinidine  Dronedarone  Flecainide  Lidocaine  Sotalol  Antiarrhythmics  Warfarin  Rivaroxaban  Heparin  Apixaban  Enoxaparin  Anticoagulants  Dabigatran etexilate |
| Procedures | Continuous positive airway pressure (CPAP) ventilation |

S**upplementary Table 3.** Incidence, hazard ratios and p-values for outcomes of interest. MACE, major adverse cardiovascular events; TIA, transient ischemic attack; MI, myocardial infarction

| Outcome | CA (n=9, 162) | No CA (n=9, 162) | Hazard Ratio | P-value |
| --- | --- | --- | --- | --- |
| MACE | 243 (2.7%) | 444 (4.8%) | 0.596 | <0.001 |
| Mortality | 84(0.9%) | 354 (3.8%) | 0.264 | <0.001 |
| Heart Failure | 63 (0.69%) | 184 (2.0%) | 0.376 | <0.001 |
| Cerebral infarction | 13 (0.14%) | 37 (0.40%) | 0.390 | 0.002 |
| Cerebrovascular disease | 46 (0.50%) | 104 (1.1%) | 0.492 | <0.001 |
| TIA | 84 (0.91%) | 111 (1.2%) | 0.822 | 0.174 |
| Ischemic heart disease | 129 (1.4%) | 245 (2.7%) | 0.581 | <0.001 |
| MI | 13 (0.14%) | 32 (0.34%) | 0.454 | 0.014 |
| Angina | 15 (0.16%) | 19 (0.21%) | 0.892 | 0.717 |
| Cardiomyopathy | 26 (0.28%) | 44 (0.48%) | 0.649 | 0.078 |

**
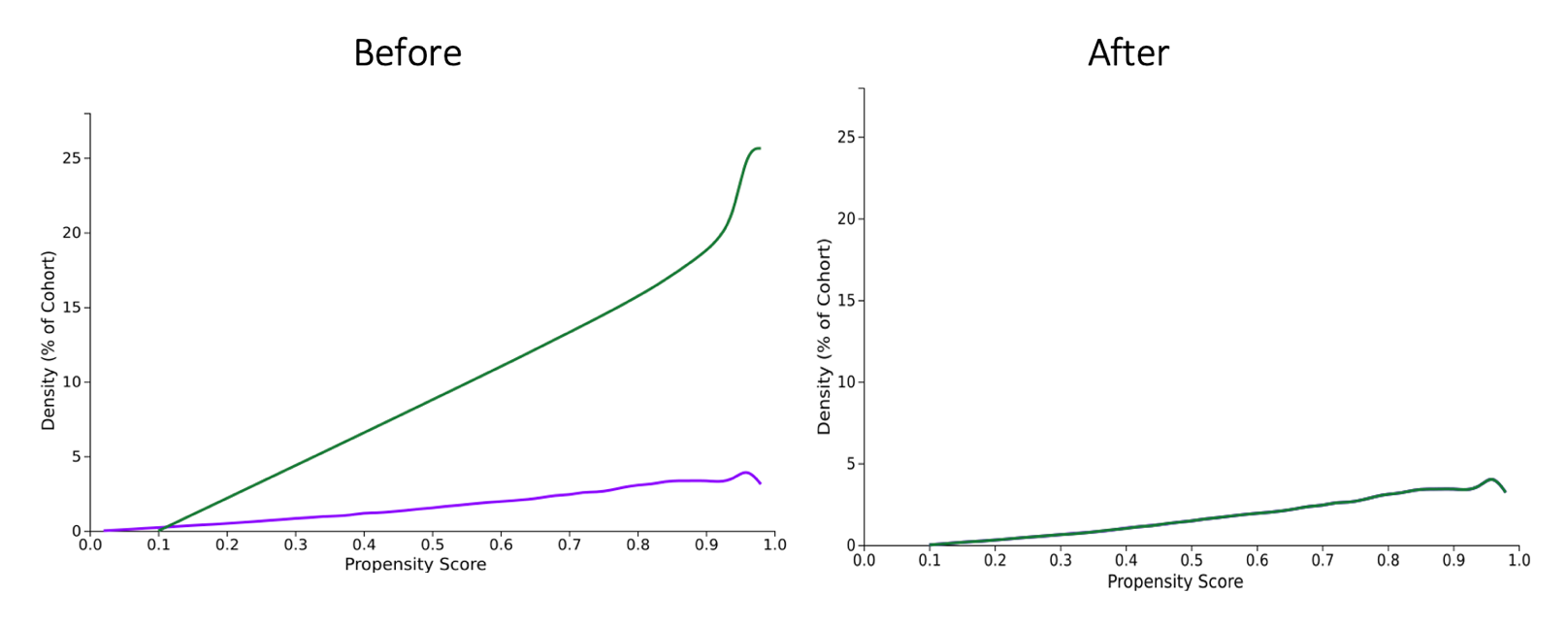
Supplementary Figure 1.** Propensity score density function - Before and after matching (cohort 1 - purple, cohort 2 - green)
